# Bodies at play: the role of intercorporeality and bodily affordances in coordinating social play in chimpanzees in the wild

**DOI:** 10.3389/fpsyg.2023.1206497

**Published:** 2024-01-16

**Authors:** Bas van Boekholt, Ray Wilkinson, Simone Pika

**Affiliations:** ^1^Comparative BioCognition, Institute of Cognitive Science, Osnabück University, Osnabrück, Germany; ^2^Division of Human Communication Sciences, School of Allied Health Professions, Nursing and Midwifery, University of Sheffield, Sheffield, United Kingdom

**Keywords:** bodily affordance, intercorporeality, chimpanzees, comparative approach, conversation analysis, evolution of language, intentionality, social play

## Abstract

The comparative approach is a crucial method to gain a better understanding of the behavior of living human and nonhuman animals to then draw informed inferences about the behavior of extinct ancestors. One focus has been on disentangling the puzzle of language evolution. Traditionally, studies have predominantly focused on intentionally produced signals in communicative interactions. However, in collaborative and highly dynamic interactions such as play, underlying intentionality is difficult to assess and often interactions are negotiated via body movements rather than signals. This “lack” of signals has led to this dynamic context being widely ignored in comparative studies. The aim of this paper is threefold: First, we will show how comparative research into communication can benefit from taking the intentionality-agnostic standpoint used in conversation analysis. Second, we will introduce the concepts of ‘intercorporeality’ and ‘bodily affordance’, and show how they can be applied to the analysis of communicative interactions of nonhuman animals. Third, we will use these concepts to investigate how chimpanzees (*Pan troglodytes*) initiate, end, and maintain ‘contact social play’. Our results showed that bodily affordances are able to capture elements of interactions that more traditional approaches failed to describe. Participants made use of bodily affordances to achieve coordinated engagement in contact social play. Additionally, these interactions could display a sequential organization by which one ‘move’ by a chimpanzee was responded to with an aligning ‘move’, which allowed for the co-construction of the activity underway. Overall, the present approach innovates on three fronts: First, it allows for the analysis of interactions that are often ignored because they do not fulfil criteria of intentionality, and/or consist of purely body movements. Second, adopting concepts from research on human interaction enables a better comparison of communicative interactions in other animal species without a too narrow focus on intentional signaling only. Third, adopting a stance from interaction research that highlights how practical action can also be communicative, our results show that chimpanzees can communicate through their embodied actions as well as through signaling. With this first step, we hope to inspire new research into dynamic day-to-day interactions involving both “traditional” signals and embodied actions, which, in turn, can provide insights into evolutionary precursors of human language.

## 1 Introduction

The field of comparative psychology investigates behavioral organization and cognition by pinpointing differences and similarities between animal species (Call et al., 2017; Bräuer et al., 2020). For many human traits, such as, for instance, hand morphology and gait, we can trace its evolutionary heritage through fossil evidence (Marzke and Marzke, 2000; Bramble and Lieberman, 2004; Pouydebat et al., 2008). However, this seems challenging for cognition and behavior because unlike physical traits, cognition and behavior do not fossilize. To counteract this problem, comparative psychologists use the comparative approach (e.g., Kamil, 1987; van Horik and Emery, 2011; Bräuer et al., 2020). In the comparative approach empirical data of multiple living species is collected to draw inferences about the behavior of extinct ancestors (Fitch, 2005, 2017). One crucial focus within comparative psychology is gaining insights into the evolution of language (Arbib et al., 2008). While language has been a topic of research for decades, its evolution still remains a mystery (e.g., Hauser et al., 2014; Pika et al., 2018; Damjanovic et al., 2022). Human language as a whole seems unique to our species. However, if we view language as a system of different layers with different phylogenetic and evolutionary origins, we may be able to trace the phylogeny and development of involved building blocks and cognitive mechanisms across the nonhuman animal (hereafter: animal) kingdom (Fitch et al., 2010; Levinson and Holler, 2014; Fitch, 2017). Comparative psychologists and scholars from other disciplines have investigated these building blocks of language using the comparative approach (e.g., Hockett and Hockett, 1960; Hewes et al., 1973; Hauser et al., 2002; Fitch, 2005; Levinson and Holler, 2014; Fitch, 2017).

Here, we adopt the line of argument (Levinson, 2006) that a fruitful way to approach questions of language evolution is to look at the primordial site in which spoken language has evolved, i.e., spontaneous mundane social interaction. The question can then be framed not so much as how did language evolve from forms of animal communication, but rather what are the possible continuities between (1) animal forms of social interaction, and (2) the practices of human social interaction, including, in particular, of talk as part of human social interaction (Schegloff, 2006). One promising aspect of interaction in this regard is sequential organization, i.e., aspects of the organization of interaction by which contributions (including utterances or non-verbal ‘moves’) are positioned relative to each other (Schegloff, 2007). This includes the phenomenon of ‘nextness’ (Schegloff, 2006), namely the inter-relationship between any particular ‘current’ utterance or move in interaction and what should/does come as the ‘next’ utterance/move. One form of sequential organization which has been shown to be present in both human and nonhuman primates is the organization of two discrete actions produced by two different participants into a sequence of actions. For example, a greeting by one participant is followed by a greeting by another (Schegloff, 2007; Mondada and Meguerditchian, 2022), or a request by one participant is followed by a response to that request (Schegloff, 2007; Wilkinson et al., 2012; Rossano, 2013b). In this paper, we explore the social interactions of one of our closest living relatives, the chimpanzee (*Pan troglodytes*) in terms of another, general, aspect of sequential organization and nextness, i.e., how an interactional activity — social play — is coordinated in terms of a series of successive ‘moves’ between the participants. To do this, we draw on research from conversation analysis (CA), and interaction research more generally into intercorporeality and bodily affordances (Meyer et al., 2017; Katila, 2018), and apply these concepts to spontaneously occuring social play interactions of chimpanzees in the wild (but see: Mondada and Meguerditchian, 2022 for an analogous approach). With this new approach, we hope to inspire further research and open up new pathways into communicative interactions and the study of language evolution.

### 1.1 The interaction engine

Recently, Levinson (2006) hypothesized that the evolution of language was possible because humans possessed a set of skills that he calls the “interaction engine.” These skills involve both the cognitive (e.g., intentionality, prosocial motivation) and the communicative (e.g., sequence organization, turn-taking organization, communication through multiple sensory modalities) domains. This interaction engine hypothesis does not represent distinct brain modules but rather should be viewed metaphorically as describing distinct principles of human interaction (Levinson, 2006; Heesen and Frohlich, 2022). While language considerably transformed human interactions (e.g., enhanced intersubjectivity; Enfield and Sidnell, 2022), the interaction engine hypothesis suggests that this distinctive set of skills involved in social interactions not only preceded the evolution of language but made language evolution possible in the first place. The evolution of these skills might have been driven by the challenges presented in group-living (Dunbar, 1993). While the entire set of skills presented in the interaction engine hypothesis might be unique to humans, single parts may have different evolutionary origins and onsets (Levinson, 2006, 2016).

One skill is the ability to act and communicate intentionally (Levinson, 2006; Heesen and Frohlich, 2022; Roberts et al., 2022). Intentionality, defined as the voluntary and goal-directed use of actions (Dennett, 1983; Townsend et al., 2017), has been a major focus in comparative research of animal communicative interactions (Graham et al., 2020; Rodrigues and Fröhlich, 2021; Schel et al., 2022) intentionality can function by switching recipient’s understanding of behavior to understanding of intention when the cognitive distraction inhibits it (Roberts and Roberts, 2022). This emphasis on intentionality, specifically in nonhuman primate gesture research, stems from studies on prelinguistic human infants (Piaget, 1952; Bruner, 1972) and early comparative studies tackling intentional use of communicative signals outside human communication (Plooij, 1978; Leavens and Hopkins, 1998; Tomasello, 2008). Indeed, intentionality has often been considered a prerequisite for communication to happen (e.g., Hobaiter and Byrne, 2011; Fröhlich and Hobaiter, 2018; but see: Roberts et al., 2014; Fröhlich et al., 2019). Because intentionality is not directly measurable (Scott-Phillips, 2015; Townsend et al., 2017), studies rely on behavioral proxies and parameters used in studies on prelinguistic human infants (Piaget, 1952; Bates et al., 1979; Bruner, 1981). These parameters involve, for instance, the gaze of the signaler and/or the recipient, adjustment to potential audiences and goal persistence (Leavens et al., 2005; Call and Tomasello, 2007; Townsend et al., 2017; Ben Mocha and Pika, 2019; Ben Mocha and Burkart, 2021; Rodrigues and Fröhlich, 2021). Using this method, studies mainly focused on signals that were accompanied by a specific set of intentionality criteria (e.g., Hobaiter and Byrne, 2011; Graham et al., 2018; but see: Roberts et al., 2014; Fröhlich et al., 2019; Aychet et al., 2021). In addition, these intentionality criteria often differed between studies (Bourjade et al., 2020; Graham et al., 2020), and are not generalizable across modalities (Rodrigues and Fröhlich, 2021). For example, the intentionality criterion *sensitivity to the attentional state of recipient* can be measured for silent-visual gestures and facial expressions but not for vocalizations or tactile gestures (Rodrigues and Fröhlich, 2021). This leads to studies *a priori* selecting a few elements of the communicative interaction for their analysis based on proxies for intentionality. Additionally, there are other elements such as gaze and relative body position that make up the communicative interaction (Wilkinson et al., 2012; Rossano, 2013a,b). While intentionality is an important component of the interaction engine (Levinson, 2006; Heesen and Frohlich, 2022), it is not the only one. For other components of the interaction engine (e.g., sequence organization or turn-taking organization), one needs to investigate all elements which are part of the interaction independent of whether they may have been produced intentionally or not (e.g., Rossano, 2013b; Mondada and Meguerditchian, 2022).

### 1.2 Conversation analysis and participants’ intentions

One field that specifically focuses on how participants achieve coordinated social interaction, while at the same time remaining relatively agnostic about a participant’s intentionality with regard to a particular element of talk or non-verbal behavior in that interaction, is conversation analysis (CA). CA has been described as the dominant approach to the study of human social interaction (Sidnell and Stivers, 2013), and has also been successfully applied to nonhuman social interactions, highlighting some components of the interaction engine (e.g., Wilkinson et al., 2012; Rossano, 2013b; Genty et al., 2020; Mondada and Meguerditchian, 2022).

CA focuses on the vocal (e.g., talk, grunts) or embodied (e.g., gesture or body movement) social practices which a participant can draw upon to produce a meaningful interactional contribution. These practices are social in that they are resources which are available to, and understood by, the members of the culture or group (Atkinson and Heritage, 1984). As such, another participant in the interaction can use their knowledge of these practices in order to make sense of a participant’s contribution and respond to it appropriately (Heritage and Atkinson, 1984; Iedema, 2003). A goal of CA is to explicate these social practices and how participants make use of them to co-create a coherent interaction on a moment-by-moment, turn-by-turn basis. Knowledge and use of these practices have been shown to underlie various features of human interaction, such as: action formation, i.e., how a contribution is designed such that it can be understood to be performing a particular communicative action (such as a request or a greeting); turn-taking organization, including how participants are able to coordinate their contributions in a timely manner; and sequence organization, which includes how sequences of actions are structured across different participants, such as how an initiating action (such as a question) produced by one participant can provide an opportunity for, or make expectable, a responding action (such as an answer) from another participant (Schegloff, 2007).

A methodological consequence of this analytic stance is that rather than the researcher speculating on what a participant intended or whether that intention was really understood by a recipient, the focus of the analysis is on observing and describing the social practices used by the participants to produce meaningful contributions and to respond to others’ contributions (Atkinson and Heritage, 1984).

### 1.3 Embodied conduct, intercorporeality, and participation

While early work in CA drew predominantly on audio-recordings of telephone calls and, as such, focused predominantly on talk, more recent work in CA and other areas of interaction research has also increasingly examined embodied aspects of social interaction (e.g., Heath and Luff, 2013; Mondada, 2016). Embodied conduct includes not only gestures, specific body movements and eye gaze behavior but also the whole bodies of participants as they engage with other participants, often in ways which involve close coordination and rapid co-adjustments as seen in activities such as sports, dancing or physical play (Goodwin, 2017; Meyer and von Wedelstaedt, 2017).

This work draws on concepts such as intercorporeality, i.e., a notion of embodied conduct that highlights how it is not possible to understand the ways in which an individual body acts or perceives within joint activities without taking into account its inter-relationship with the bodies of these others which are simultaneously acting and perceiving (Merleau-Ponty, 1962; Meyer et al., 2017). As Meyer et al. (2017, p. xviii) put it in relation to the human body, from an intercorporeality perspective, the human body is “*constituted* by its corporeal relations and interactions with other human or animal bodies” (emphasis in original).

In understanding how such joint activities can be successfully achieved, the notion of bodies as instruments or ‘signalling devices’ (Meyer et al., 2017) expressing in physical form the products of previously conceived intentions appears to have limited application. Rather, from an intercorporeal perspective, intentionality can be conceptualized as ‘interactionally emergent rather than causally prior to action’ (Meyer and von Wedelstaedt, 2017, p. 12; see also Heesen et al., 2017; Bangerter et al., 2022).

In summary, research on intercorporeality focuses on the inter-relations between bodies and their actions rather than on individual bodies and actions. While work drawing on the notion of intercorporeality would appear to have much to offer studies of animal communication and animal social behavior, the application so far has been limited (but see, e.g., Due, 2023, on intercorporeality in an interspecies interaction).

Examining intercorporeality can be one way of investigating how participants participate together in a social activity. The notion of participation has been heavily influenced by the work of Goffman, including his concept of a participation framework (Goffman, 1981). Goffman’s concept primarily concerned the relationship between human participants in an encounter which includes talk, such as a multiparty conversation, and focuses on the different types of roles that speakers and hearers can be inhabiting at a particular point in time within the interaction. More recently, work on participation and participation frameworks has broadened in various ways, including moving away from quite a static picture of participant roles to a focus on more dynamic features of how participants collaborate together in the co-construction of activities through shifting embodied participation frameworks (Goodwin and Goodwin, 2004; Goodwin, 2007) as well as being extended to nonhuman participants, such as chimpanzees engaged in social play (Heesen et al., 2017) or bonobos (*Pan paniscus*) in joint-travel initiations (Rossano, 2013b). It is this broader conception of participation and participation frameworks that we will draw on here.

### 1.4 Bodily affordances and signals

One feature of intercorporeality concerns how bodies engaged in a collaborative activity (e.g., acrobatics: Brümmer and Alkemeyer, 2017) provide affordances for other bodies to produce certain actions. The notion of affordances comes from Gibson (1986) who discussed the affordances provided by both inanimate and animate objects. Our focus here will be primarily on the affordances provided by animate objects (people and animals), and specifically on how the affordances provided by chimpanzees’ bodies during contact social play provide opportunities for responsive action and thus facilitate the coordination of that play.

The nature and use of such *bodily affordances* have been explored in human interaction. For example, Katila (2018), in a study of intercorporeal formations between mothers and their (2 to 3 year old) toddlers, showed how different corporeal formations between mother and child provided different affordances. For example, a ‘nested tactile arrangement’ where, for instance, the child nestled in the mother’s lap, afforded continual tactile communication between mother and child, in that the mother could at any moment rock, stroke or tickle the child. A ‘distal tactile arrangement’, on the other hand, where the mother and child were physically close but not in constant physical contact, afforded both bodies greater independence, such as the mother being able to more easily interact with others.

A focus of our analysis will be how a bodily affordance provided by one chimpanzee during contact social play can provide an opportunity for responsive action by another chimpanzee, thus facilitating the fluent moment-by-moment co-construction of this play activity. This sequential organization of (1) what the bodily affordance offers and (2) how another chimpanzee responds to that affordance is somewhat analogous to sequence organization in talk-in-interaction (Schegloff, 2007) where a verbal action such as a question by one participant sets up a ‘next move slot’ for a responsive action (an answer) by another (see also Hutchby, 2001; Withagen et al., 2012, on different ways in which affordances can provide opportunities for action).

Bodily affordances differ from more “traditional” signals used in the comparative study of communication (King, 2004). Within the study of animal communication, signals are commonly divided into three categories: vocalizations, gestures and facial expressions (Slocombe et al., 2011; Liebal et al., 2013). Signals are seen as distinct units of information which are send from a signaler and affect the behavior of the receiver. They have been traditionally studied as part of the information-processing paradigm (Shannon, 1948; Seyfarth et al., 2010; Wheeler et al., 2011). They are classified based on their form, and different signals might lead to the same behavioral change or the other way around (e.g., Call and Tomasello, 2007; Hobaiter and Byrne, 2014). While some bodily movements, such as gestures, are studied in this paradigm, bodily affordances better fit a different paradigm, that of dynamic system theory (Shanker and King, 2003; King, 2004; King and Shanker, 2016). However, this does not mean that signals and bodily affordances cannot be studied simultaneously. Bodily affordances are part of any social conduct involving bodies. On top of this bottom layer analysis of interacting bodies, signals (intentionally produced or not) can be added for increased complexity. Here, we want to show how bodily affordances can add to the comparative study of communication in conjunction with “traditional” signals.

### 1.5 Social play in chimpanzees

In this paper, we wish to draw on the concepts of intercorporeality and bodily affordances to explicate how chimpanzees can collaboratively and fluently produce contact social play activities together. Contact social play activities, such as play biting, can be contrasted with non-contact social play activities, such as chasing (Caro, 1995). Joint activities such as these can present a puzzle in terms of how animals can co-produce them with such apparent ease and fluency, given that they involve improvisation, rapid coordination and fast adjustments (Pellis and Pellis, 1996; Palagi, 2006). Play contexts have long been an object of interest for comparative psychologists for various reasons. For instance, in regard to human infants, playing is crucial for the development of metacognitive skills such as planning, reflection, and self-regulatory behaviors (Whitebread et al., 2009), and is linked to language acquisition (Tomasello and Rakoczy, 2003; Orr, 2021). Concerning other animals, playing is common across several taxa (Bekoff and Byers, 1998). Additionally, this behavioral domain has also been suggested to be a useful platform to study both the evolution of shared intentionality (Heesen et al., 2017) and joint commitment as a process (Heesen et al., 2021a; Bangerter et al., 2022; Rossano et al., 2022). As Heesen et al. (2017, p. 1) note about social play: ‘One aspect that has recurrently fascinated scholars is its complex, cooperative nature that requires substantial on-the-fly coordination and improvisation (Bekoff and Allen, 1998; Bekoff, 2001; Palagi, 2006), in comparison to other social activities like grooming or sex that involve more stereotyped activity-specific patterns’. Being a dynamic, as well as a very common, form of interaction, social play forms an ideal context to study the different components of the interaction engine (Heesen et al., 2017).

However, due to its dynamic nature playing is difficult to analyze using a “traditional” approach, as possible underlying intentionality is hard to assess. Additionally, playing often comprises many bodily movements that do not readily fall under “traditional” signal categories. Therefore, while playing is of interest for many reasons, it has received relatively little research attention in studies of animal communication (but see: Pika et al., 2003, 2005; Palagi, 2008; Heesen et al., 2017). Existing studies into playing interactions have focused on specific signals (Pika et al., 2003, 2005; Palagi, 2008; Genty and Byrne, 2010; Mancini et al., 2013), specific phases (Flack et al., 2004; Fröhlich et al., 2016a), or described the whole playing interaction (Palagi, 2006; Heesen et al., 2017, 2021a,b). Most studies have investigated species living in captivity (but see: Fröhlich et al., 2016a).

In our analysis, we specifically focus on data of spontaneous interactions of Eastern chimpanzees (*Pan troglodytes schweinfurthii*) living in a habituated community in their natural environment, the Kibale National Park in Uganda. The communicative repertoire and usage of chimpanzees have been extensively studied (e.g., Goodall, 1986; Hobaiter and Byrne, 2011; Tomasello and Call, 2018; Crockford, 2019). However, to better understand quick, dynamic joint activities such as contact social play, traditional approaches focusing on communicative signals are not expedient. For such activities, we suggest to adopt and expand analyses drawing on concepts and methodologies from work into intercorporeality, such as that of bodily affordances. Such an approach can provide insights into how chimpanzees and possibly other animal species co-produce these rapidly changing joint activities through particular forms of communication and co-ordinated action.

## 2 Materials and methods

Data collection used for the current analyses occurred from February–September 2021 as part of a bigger project studying the development of turn-taking interactions in chimpanzees in the wild. Data was collected from two neighboring groups (central group, *n* = 120; western group, *n* = 85) of the Ngogo community, Ngogo Chimpanzee Project, Kibale National Park, Uganda. Detailed description of the Ngogo field site and study area can be found in Struhsaker (1998) and Butynski (1990).

### 2.1 Data collection

A total of 22 mother-infant dyads (9 females, 13 males) were followed on 155 days between 7:00 AM and 17:30 PM. This method resulted in a total of 139 h of video recordings with 2,723 interactions of which 825 interactions involving playing (i.e., two or more individuals engaging in playing without a break of more than 30 s; Koski et al., 2007; Newton-Fisher and Lee, 2011; Roberts et al., 2012). The recordings were made using a digital camera (Sony AX100E 4 K) and a directional microphone (Sony shotgun ECM-CG60) to capture all visual and audible acts produced in the interactions. Data was collected following the sampling rule of focal-animal sampling focusing on the infant with a recording rule of continuous recording when the visibility allowed for it (Altmann, 1974; Martin and Bateson, 2007). The ages of the infants ranged from 3 to 46 months (median: 21 months).

For the purpose of the present paper, we selected four different videos from the bigger dataset based on the following rationale: They (a) were representative of a playing session in the dataset; (b) were characterized by a high level of visibility, meaning both participants, their communicative signals and movements could be clearly seen; (c) showed instances of bodily affordances; and (d) involved different physical arrangements (e.g., nested arrangement, one individual hanging from a branch in front of/above the other individual) and, in particular, play types (e.g., play biting, tickling, falling on the other individual). Each video contained one playing session. From these playing sessions, we extracted one or multiple episodes to highlight different ways interactants could, initiate, end, or maintain a joint contact play. More detailed information concerning the individuals involved in the selected videos, as well as which extracted episodes relates to which video, can be found in the Supplementary Table S1. A video, of each extracted episode can be found in the Supplementary Videos S1–S8.

### 2.2 Definitions

The episodes described below are from videos that all involve social play interactions, specifically contact social play between an infant and an adult individual. Contact social play was defined as at least two individuals engaging in a social interaction containing playful behaviors which involve chimpanzees’ bodies touching each other in some manner (e.g., play biting, tickling) and signals associated with play (e.g., play face, slap other; Flack et al., 2004; Fröhlich et al., 2016a). For playing to commence, both participants needed to actively engage in the playing interaction. Every example contains one playing session. However, similar to grooming (Goodall, 1986), playing is often not a continuous activity and one playing *session* can contain multiple playing *bouts* interspersed with breaks. A play bout ended when one of the participants ceased its play behavior. We defined the end of a play session when play behavior stopped for more than two minutes (Heesen et al., 2021a,b). However, it should be noted that using a time criteria to distinguish between the end of a playing bout (interruption) and a playing session (true ending) is, ultimately, arbitrary.

To refer to distinct signals used, we adopted terms from the existing literature on chimpanzee communication (Nishida et al., 1999; Parr et al., 2005; Call and Tomasello, 2007; Hobaiter and Byrne, 2011) and denoted them in capitals (Table 1).

**Table 1:** Definitions of signals used in the playing sessions analyzed.

Signals	Definition
EXTEND HAND	One arm is reached out to the recipient with the palm held vertically or upwards and the fingers in an open position.
GRAB	One hand is firmly closed over a part of the recipient’s body.
HEAD SHAKE	The head is repeatedly moved back and forth (side to side or vertical).
MOUTH STROKE	The signaler’s palm or fingers are repeatedly run over the mouth area of the recipient.
PLAY FACE	The face is relaxed with mouth open exposing the bottom teeth. The upper lip may be raised slightly exposing the upper teeth.
SLAP OTHER	The signaler hits the body parts of the recipient forcefully with the palm of the hand.

### 2.3 Analysis of data

The analysis of contact social play activities in chimpanzees focused on the behavioral means through which adult and infant chimpanzees together negotiated and achieved the ‘on-the-fly coordination’ of play mentioned by Heesen et al. (2017). Specifically, we described these practices (Meyer and von Wedelstaedt, 2017; Mondada and Meguerditchian, 2022) in terms of signals and, in particular, of the inter-relationship between the two chimpanzee’s bodies (including within their material context, such as the tree sapling a chimpanzee is sitting on or hanging from) through which chimpanzees communicate with each other and collaborate together to initiate, end and maintain episodes of contact social play. We do not make a strong distinction between practical corporeal activity (such as an infant climbing into its mother’s lap) on the one hand, and communication on the other. As Katila (2018, p. 5) notes (in relation to bodies in contact with each other) “while producing practical actions in tactile intercorporeality, bodies are inevitably communicating their moment-by-moment unfolding actions and action intentions to each other.”

The embodied conduct and postural alignment of a chimpanzee can provide an affordance (Gibson, 1986) to another chimpanzee. This means that the body can be presented to another individual in such a way that it affords the other individual an opportunity to do something in relation to that body with their own body. That is, these corporeal behaviors and postures may not constitute solely individualistic conduct, but rather can have a social and communicative aspect, i.e., making the body available, or proffering it (with whatever level of conscious awareness) to the other in such a manner that it can be used by that other in a certain way. The analysis focused on the nature of these bodily affordances and the type of sequential organization they make possible through providing a ‘next move slot’. Here, another chimpanzee has the opportunity to align with the move constituted by the bodily affordance and take forward the activity which is underway.

We primarily, but not exclusively, drew on examples from three different types of adult-infant play, i.e., contact social play sessions where the play primarily consisted of:

- play biting of the infant by the adult,
- tickling or grabbing of the infant by the adult, and
- play involving the infant falling onto the adult and the adult patting or grabbing the infant.

A focus of the analysis is the interactional and sequential process by which the contact social play activities are co-created and negotiated on a moment-by-moment and move-by-move basis between the chimpanzees (see also Genty et al., 2020). This process has a number of stages which the chimpanzees must proceed through together for a bout of play to be initiated and maintained. At the same time, it highlights the contingency of the activity in that despite one chimpanzee engaging in play behavior or play communication, there is a reliance on the other chimpanzee to co-engage in order for contact social play to start or be maintained.

The stages of the process we observed and analyze below are as follows: In each case, we signpost relevant examples (presented as ‘Episode 1’ etc.) that will be discussed in the Results section.

#### 2.3.1 Establishment of a participation framework which affords contact social play

One focus of the analysis was to examine how chimpanzees collaboratively moved from being engaged in a non-play activity to that of a contact social play activity. A prerequisite for contact social play is that the chimpanzees align their bodies to each other in such a way that a participation framework (Goodwin and Goodwin, 2004; Rossano, 2013b) is achieved where contact social play is possible (Episodes 1 and 2). Each of the three main types of play we analyzed could be seen to involve different participation frameworks and bodily alignments. For example, in the case of play biting, the relevant part of the adult’s body was its mouth, and a participation framework had to be jointly achieved such that a part of the infant’s body was close to the adult’s mouth. This stage is similar to the pre-entry phase of joint activities described by Heesen et al. (2017) and Genty et al. (2020).

#### 2.3.2 Initiation of contact social play

This stage can overlap to some extent with what Heesen et al. (2017) and Genty et al. (2020) term the ‘entry’ phase. Once a participation framework (which affords play) is present, a chimpanzee can make an initiating move, hereafter referred to as MOVE 1, toward contact social play by signaling or, more commonly in our data, engaging with the other’s body in a play-related way, e.g., by play-biting (Episodes 1 and 2).

This bodily behavior can set up a context such that the other chimpanzee can signal or act in a manner which can be seen as an *aligning responsive move* (Schegloff, 2007), hereafter referred to MOVE 2. That is, the responding chimpanzee can take forward the activity that the earlier action initiated. For instance, chimpanzee A produced a play-related activity and chimpanzee B responded with a play-related activity indicating that contact social play involving the two chimpanzees has been successfully initiated (Episodes 1 and 2). This responding move implicitly displays an understanding of the prior move (Schegloff, 2007; Mondada and Meguerditchian, 2022) as willingness to engage in social play and to go along with the initiated activity of the first actor.

Alternatively, the stage of successful joint initiation may not be reached. This can be because neither chimpanzee moves to initiate play despite being within a participation framework that affords it. It can also be because despite one chimpanzee initiating a move into play behavior, the other chimpanzee does not reciprocate. This can take the form of either not engaging with chimpanzee A (Episode 3), or engaging, but in the form of a non-play activity, such as grooming (Episode 4).

#### 2.3.3 Ending of a contact social play bout or session

A contact social play bout can be ended by one chimpanzee transitioning the interaction into a non-play joint activity (Episode 5) or physically disengaging from the play activity (Episode 6). This stage is analogous with the ‘exit’ phase of joint activity (Heesen et al., 2017; Genty et al., 2020). In our data, this practice of disengaging from the play activity (with perhaps a transition to another, non-play, joint activity) is more common than producing gestural signals to communicate about the possible ending of a play bout (compare, e.g., Genty et al., 2020). When a play bout has ended, an analytic question can be whether one or both chimpanzees act to display that they are providing an opportunity for another episode of play (e.g., through providing an affordance for further play: Episode 8). This phase of the activity would appear to overlap with what has been referred to as ‘suspension’ and ‘reengagement after interruption’ (Heesen et al., 2017; Genty et al., 2020).

#### 2.3.4 Maintenance of contact social play

If contact social play has been initiated by chimpanzee A (MOVE 1), and this initiation has been reciprocated by chimpanzee B (MOVE 2), then the jointly produced activity is underway. The next stage of the interactional process is whether, and how, it may be taken forward and maintained by chimpanzee A and/or chimpanzee B in a moment-by-moment fashion (Episode 7). Maintenance of play beyond the two-move initiation sequence is dependent on a subsequent, play move (hereafter referred to as MOVE 3) being produced by one or both chimpanzees in this sequential context, rather than the alternative ‘move’ of either disengagement or engagement in non-play behavior which would constitute an ending of the play bout. When such a sequence of moves is enacted, the chimpanzees’ activity can be seen to constitute a particular form of interactional alignment, i.e., where two bodies are displaying ‘agreement’ in relation to each other regarding what the activity underway is and a willingness to jointly co-construct it (*cf.* Heritage and Atkinson, 1984; Schegloff, 1992 on the sequential architecture of intersubjectivity). This stage broadly aligns with what Heesen et al. (2017) and Genty et al. (2020) term the ‘continuation’ of the joint activity.

## 3 Results

Here we present findings of how a participation framework for contact social play is brought into being by infant-adult chimpanzee dyads, and how the play activity can be (i) initiated, (ii) ended, and (iii) maintained. We highlight the central role of the intercorporeal relations between the two bodies and, in particular, the way in which the affordances for play-related activity provided by a chimpanzee’s body can be responded to with play activity by the other chimpanzee. The contingent nature of contact social play as an activity, which is interactionally co-constructed on a move-by-move basis by the two chimpanzees, is highlighted by the inclusion of examples where play does not happen, either through disengagement or through engagement in a non-play activity. The results are structured in terms of the following sections:

Section 3.1: Establishing a participation framework and initiating contact social play [with sub-sections on unilateral initiations which lead to joint initiations (Section 3.1.1) or unilateral initiations which do not (Section 3.1.2)].
Section 3.2: Ending contact social play (with sub-sections on endings brought about by a chimpanzee engaging in a joint non-play activity or stopping play-related physical engagement).
Section 3.3: Maintaining contact social play (with sub-sections on maintaining a play bout and maintaining a play session).

For each episode an accompanying video can be found in the Supplementary Videos S1–S8.

### 3.1 Establishing a participation framework and initiating contact social play

#### 3.1.1 Unilateral initiations of social play which lead to joint initiations

Since play is a cooperative activity, it takes both chimpanzees to coordinate their behavior for a bout of play to have been successfully initiated. We will show here how the initiating of joint play involves:

- the collaborative achievement of a participation framework which affords an opportunity for contact social play, and
- an initiating move into contact social play by one chimpanzee, followed by an aligning responsive play move by the other chimpanzee.

##### 3.1.1.1 Initiating contact social play involving play biting

**Figure 1:**
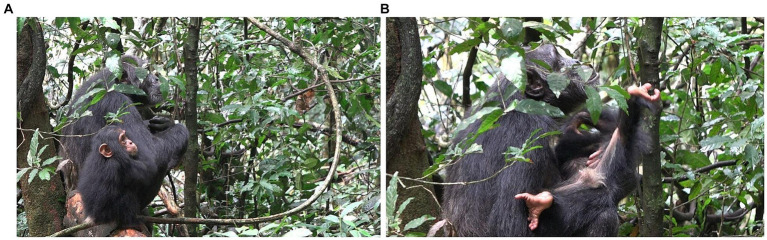
Episode 1: **(A)** Side-by-side arrangement preceding contact social play, **(B)** creation of participation framework between Lecter and Penelope.

In Episode 1, Lecter is sitting next to his mother, Penelope, on the trunk of a fallen tree, in a side-by-side arrangement without body contact (Supplementary Video S1). There are calls and screams of other individuals around, implying some tension in the surrounding group. Penelope can be seen to be paying attention to the vocalizations, including visually, focusing her gaze in the direction the sounds are coming from. Lecter gazes in the same direction as Penelope (Figure 1A).

After ~15 s, during which Lecter and Penelope stay in the same position, Lecter engages with Penelope’s body by turning toward her and performing two SLAP OTHER gestures in rapid succession. Directly after these gestures, Penelope extends her arms out in front of her a little more than they were, and Lecter climbs into her arms in a nested arrangement. Penelope supports him as he then wriggles around in her arms, with his abdomen facing up toward her face (Figure 1B). The intercorporeal positions of Penelope and Lecter at this point constitute a participation framework which provides an affordance for contact social play, most obviously by Penelope lowering her face and play biting Lecter. This participation framework is a collaborative achievement brought about by the activity both of Penelope (through presenting her arms for Lecter to climb into, and physically ‘catching’ and supporting him when he does so), and Lecter (through climbing into this position where he presents his abdomen to Penelope). Contact social play does not, however, take place during this time; Penelope continues to keep her head raised, gazing around her, rather than lowering it to Lecter’s body. After ~10 s in this nested arrangement, Lecter slides down out of this position, decreasing the affordance for contact social play.

**Figure 2:**
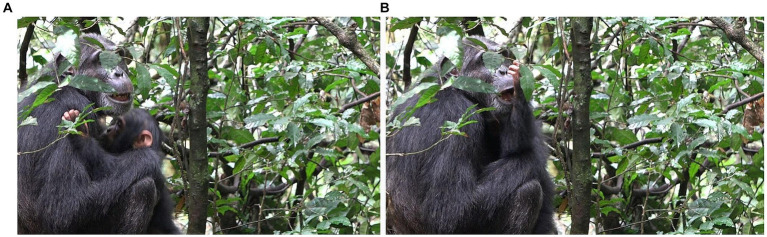
Episode 1: **(A)** Recreation of participation framework between Lecter and Penelope, **(B)** initiating move (MOVE 1) of Lecter (MOUTH STROKE gesture).

However, ~3 s later, with Penelope still retaining the same body position, Lecter stands up. With his body now vertically against Penelope’s, he swings his left arm up toward her right shoulder, producing a SLAP OTHER gesture on her shoulder. As his arm goes past Penelope’s mouth, Penelope turns her face to her right toward the moving arm and opens her mouth (although no contact takes place between Lecter’s hand and Penelope’s mouth), reacting with a PLAY FACE and thus signaling readiness for play (Figure 2A). At this point, therefore, a participation framework between the two bodies has again been collaboratively produced which affords contact social play (for example, where Penelope could play bite a part of Lecter’s body, such as his moving arm/hand). In addition, both infant and adult have signaled a readiness for play (Lecter’s arm movement/SLAP OTHER, and Penelope’s PLAY FACE).

**Figure 3:**
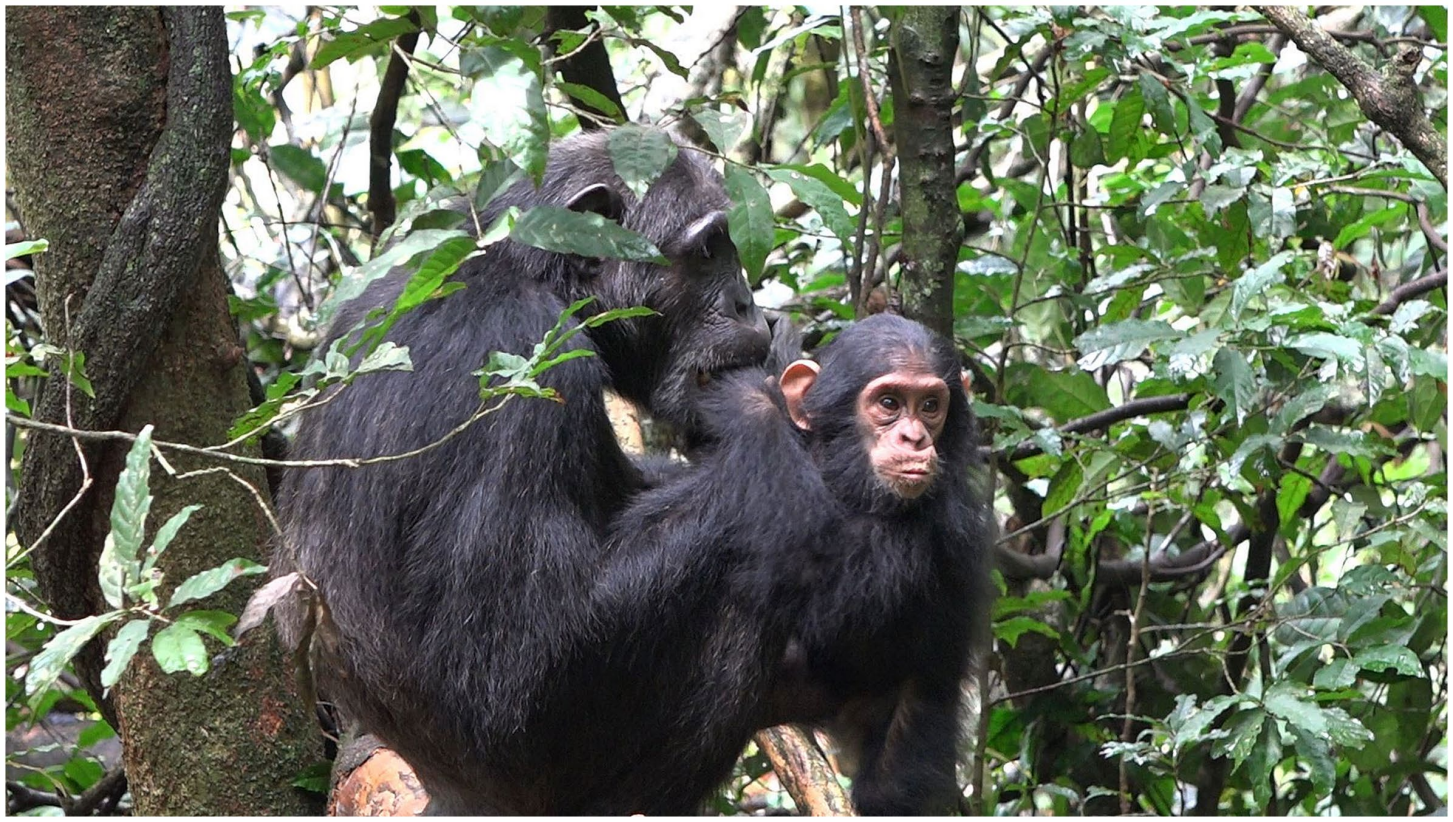
Episode 1: Responding aligning move (MOVE 2) of Penelope (play bite) jointly initiating contact social play.

While a participation framework for possible play is now in place and both chimpanzees have produced possible play-related signals, no joint play has yet happened. However, at this point Lecter brings his left hand back in front of Penelope’s open mouth and holds it there in a MOUTH STROKE gesture, providing an affordance to Penelope for play biting his hand. This contact constitutes a unliteral initiating move (MOVE 1) into possible contact social play (Figure 2B). Penelope responds by keeping her mouth open against Lecter’s hand (although not yet play biting) and, when Lecter temporarily withdraws his hand and then raises it again, Penelope shifts her open mouth to where Lecter’s hand is and closes it on his hand, taking it within her mouth and now clearly play biting (MOVE 2: Figure 3). Both chimpanzees have now collaborated to initiate contact social play.

##### 3.1.1.2 Initiating contact social play involving falling and patting

Prior to the contact social play session focused on here (Episode 2), the infant, Lootus, has been engaged in solitary play, climbing up a sapling next to an adult, Rollins, while her mother, Leigh, is close by in the background (Supplementary Video S2).

They are in the middle of a group and Lootus has been involved with several playing interactions in the minutes leading up to this episode that involved other juveniles and one infant but not her mother or Rollins. As Lootus climbs the sapling, Rollins is neither turned toward Lootus nor gazing at her. However, when Lootus climbs high enough for the sapling to bend down, with Lootus hanging on it, it comes down in front of Rollins, and Lootus lands on the ground beside Rollins. This may be an attempt by Lootus to engage Rollins in play, and indeed immediately following Lootus swinging down on the sapling, Rollins performs a small HEAD SHAKE and lies down, opening up his chest and creating an affordance for playing (for example, for Lootus to swing down onto him, as happens later in the episode). Lootus, however, continues to engage in solo play on the sapling, despite Rollins maintaining the same position and thus continuing to provide an affordance for play. After ~25 s of solo play, Lootus climbs up the sapling and falls in the direction of Rollins, landing in front of him. Lootus hangs close to Rollins for ~7 s after which she climbs back up the sapling. Due to foliage between the chimpanzees and the camera it is not possible to see if there is physical contact or contact play between Lootus and Rollins.

**Figure 4:**
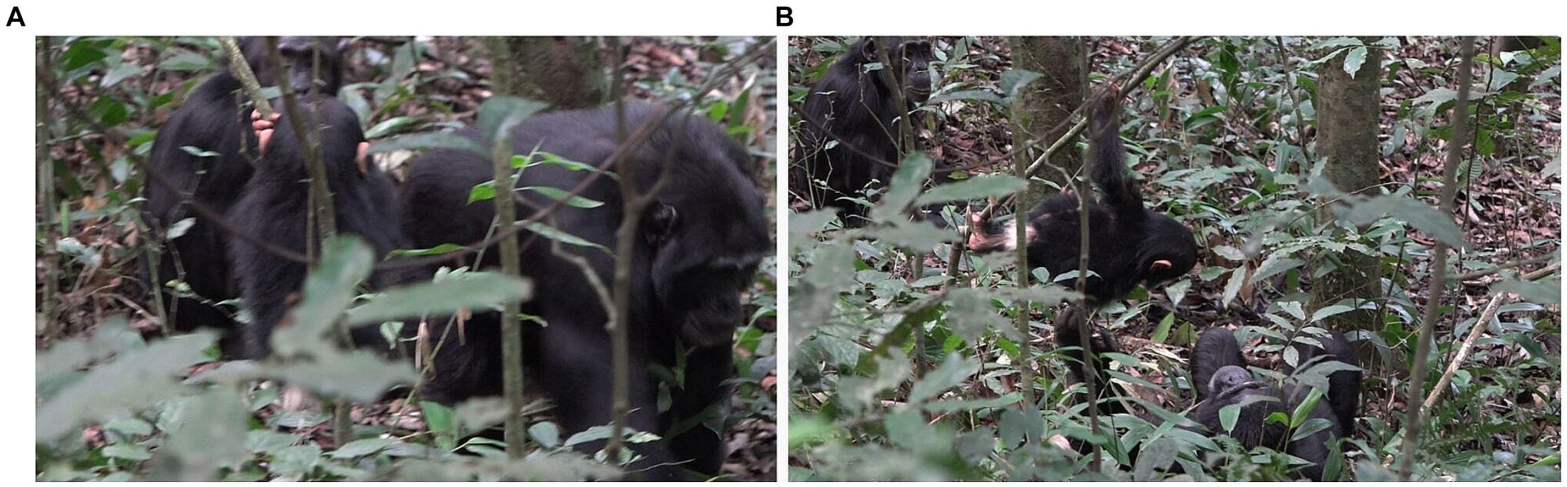
Episode 2: **(A)** Rollins (right) moves over creating a new participation framework, **(B)** creation of participation framework between Lootus and Rollins.

As Lootus climbs back on the sapling and it bends backwards over Rollins he stands up and moves across to a point 180 degrees (right side of frame) from where he previously was lying (Figure 4A). This new point is in the trajectory of where the sapling will land based on the way in which Lootus is currently hanging on it and bending it back. While Rollins is in the process of lying down in this new place, Lootus climbs up the sapling and swings on it toward Rollins. As Lootus approaches him from above, Rollins produces an EXTEND HAND gesture with his left hand, opening up his body and providing an affordance for play, in the form of facilitating Lootus to fall on to him, with Rollins catching her with his extended hand (Figure 4B). A participation framework for play, in the form of Lootus being able to fall on to Rollins, and Rollins then being able to pat, tickle or play bite Lootus, has now been collaboratively set up by the two chimpanzees.

**Figure 5:**
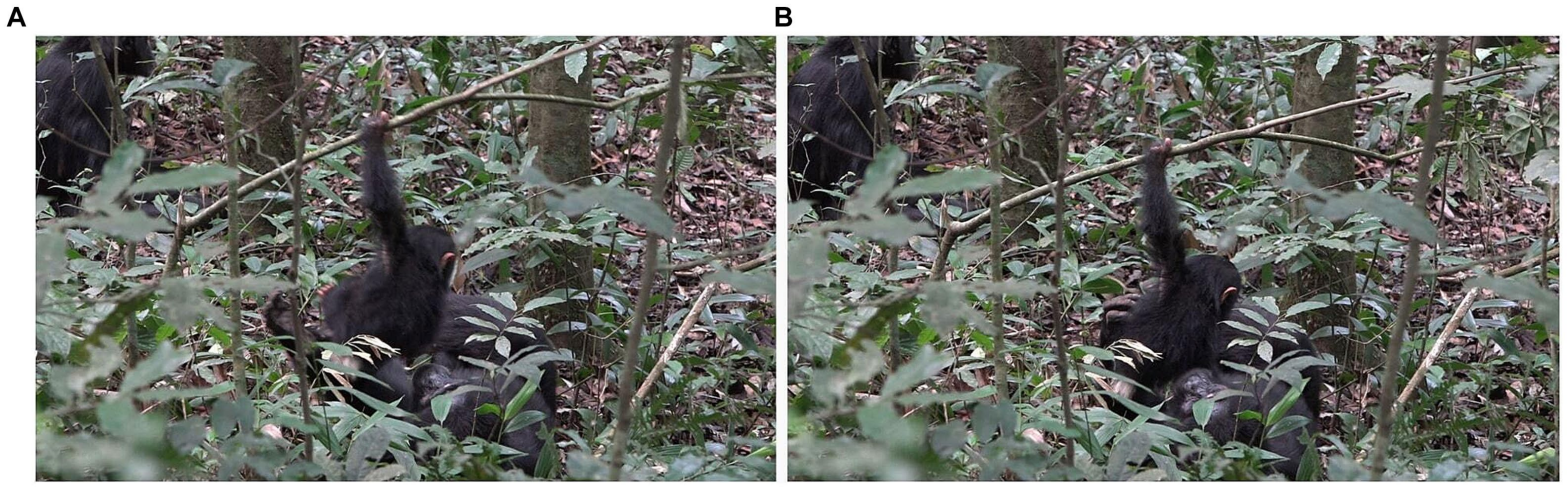
Episode 2: **(A)** Initiating move (MOVE 1) of Lootus (swinging onto Rollins), **(B)** responding move (MOVE 2) of Rollins (EXTEND HAND) initiating contact social play.

With the participation framework for possible contact social play now established, Lootus swings onto Rollins’ body, initiating a play bout (MOVE 1; Figure 5A). As Lootus holds herself in position, hanging from the sapling above the lying Rollins, Rollins responds to Lootus’ initiating move in the form of patting her (MOVE 2; Figure 5B). With this aligning move by Rollins, contact social play has now been jointly initiated. This alignment lasts for ~8 s.

#### 3.1.2 Unilateral initiations of social play which do not lead to joint initiations

Just as a chimpanzee can respond to an initiating play move by another chimpanzee with an aligning play move of their own, resulting in a bout of contact social play being jointly launched (as seen in Sections 3.1.1.1 and 3.1.1.2), it is also possible for a chimpanzee to stop a possible contact social play bout in its tracks by not responding with an aligning play move. We will now examine two types of ‘failed’ initiations, where despite one chimpanzee initiating play, the other chimpanzee does not align, and no contact social play occurs at that point. In the first type, following an initiation by chimpanzee A, chimpanzee B does not physically engage with chimpanzee A (e.g., by touching them), with the result that no aligning responding play move is produced. In the second type, chimpanzee B does physically engage with, chimpanzee A but uses the current participation framework to engage with them in an activity other than play.

##### 3.1.2.1 Chimpanzee B not engaging with chimpanzee A

**Figure 6:**
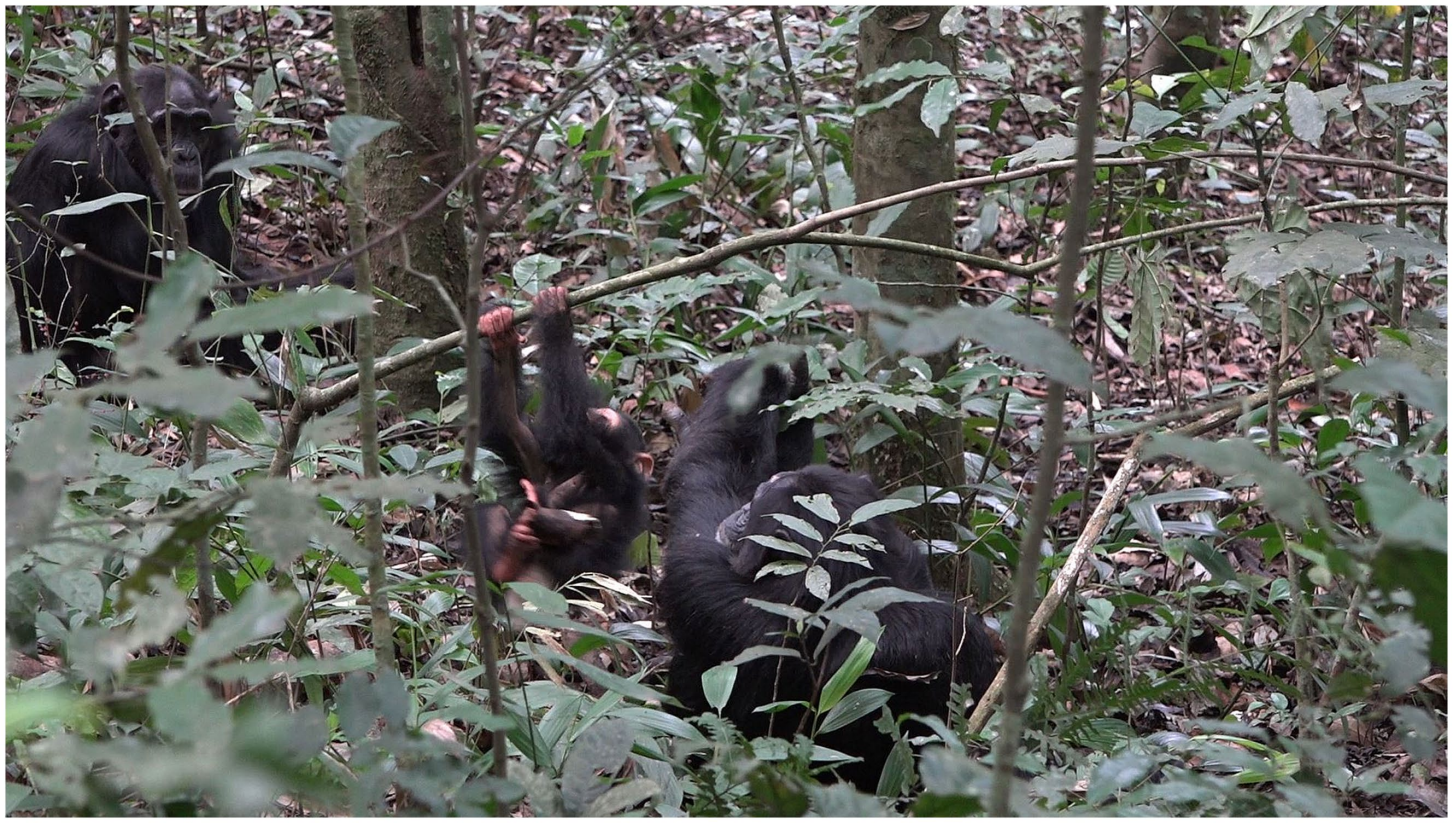
Episode 3: Rollins ending affordance for play by grabbing his left foot and “blocking” Lootus swinging onto him.

Episode 3 is from the same play session involving Rollins and Lootus seen in Episode 2 (Section 3.1.1.2) (Supplementary Video S3). This episode occurs around 3.5 min after that seen in Section 3.1.1.2, and in the interim there has been a series of play bouts between the two. Just prior to the episode, Rollins and Lootus have been engaged in a brief bout of play similar to that seen in Episode 2 (i.e., Lootus swinging onto Rollins, and Rollins then patting Lootus). Following this, Lootus climbs away and the current playing bout has stopped. As we join the episode, Rollins shifts his body posture in a significant way; he sits up a little and grabs his left foot with his left hand (Figure 6), while looking at Lootus, who is swinging from the sapling. This new posture stops the affordance for the type of play that Lootus has been engaged in with Rollins over several bouts of play during this play session (swinging on to him from his left), and, in effect, creates a barrier between Lootus and the rest of Rollins’ body. Despite the participation framework now not being conducive for the same type of play, Lootus tries to (re)engage Rollins in play in the same way as before; she swings toward him, and, when she can no longer land on Rollins’ body, she pursues her attempt at play by twice performing a MOUTH STROKE gesture and an EXTEND HAND gesture, touching Rollins on the shoulder (MOVE 1). Rollins turns his head to Lootus, but does not change his posture to facilitate play, and does not physically engage with Lootus. As such, despite this initiating play move by Lootus (MOVE 1), there is no aligning move by Rollins and contact social play is not re-initiated. After ~10 s Lootus gives up her attempt at engaging Rollins in play; she goes to her mother, after which they both leave the scene.

##### 3.1.2.2 Chimpanzee B engaging with chimpanzee A but with a non-play activity

In Episode 4, Louis, an infant, is lying next to his mother, Sabin, facing her, with Sabin’s right arm around his body (Supplementary Video S4). Louis is potentially nursing but it is not possible to confirm this from the camera angle. A male, Williams, approaches them both and starts to groom Sabin on the left side opposite from where Louis is lying. During the duration of this episode, Sabin does not move and takes no active part.

**Figure 7:**
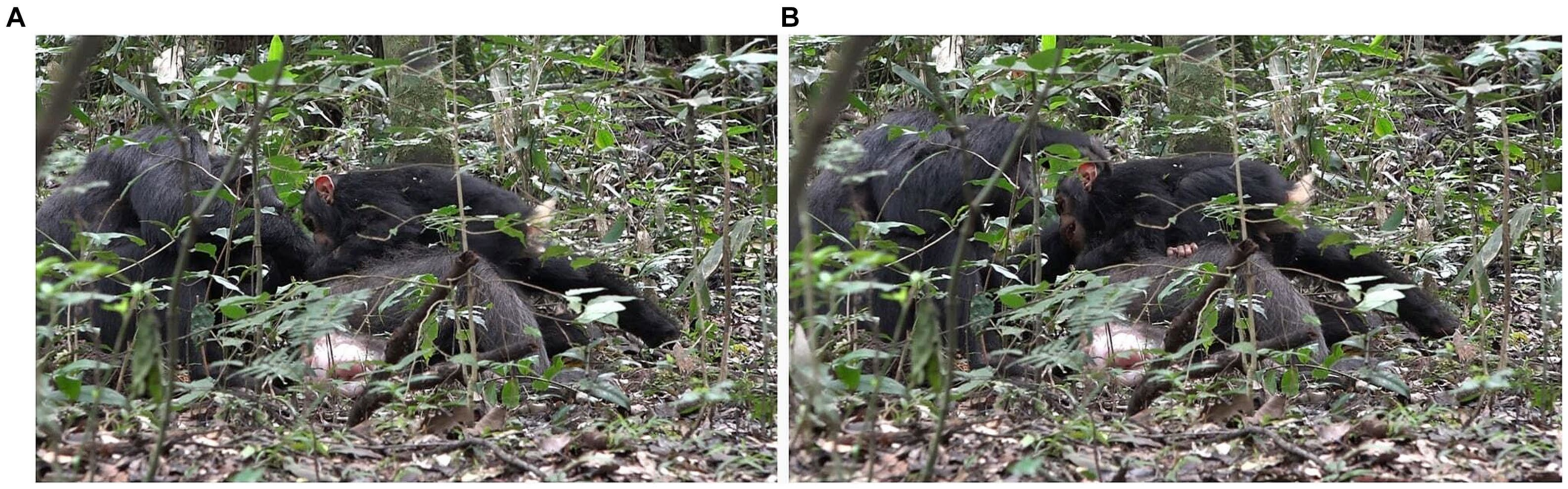
Episode 4: **(A)** Creation of participation framework between Williams (Left) and Louis (top right), **(B)** initiating move (MOVE 1) of Louis (PLAY FACE and lowered head).

**Figure 8:**
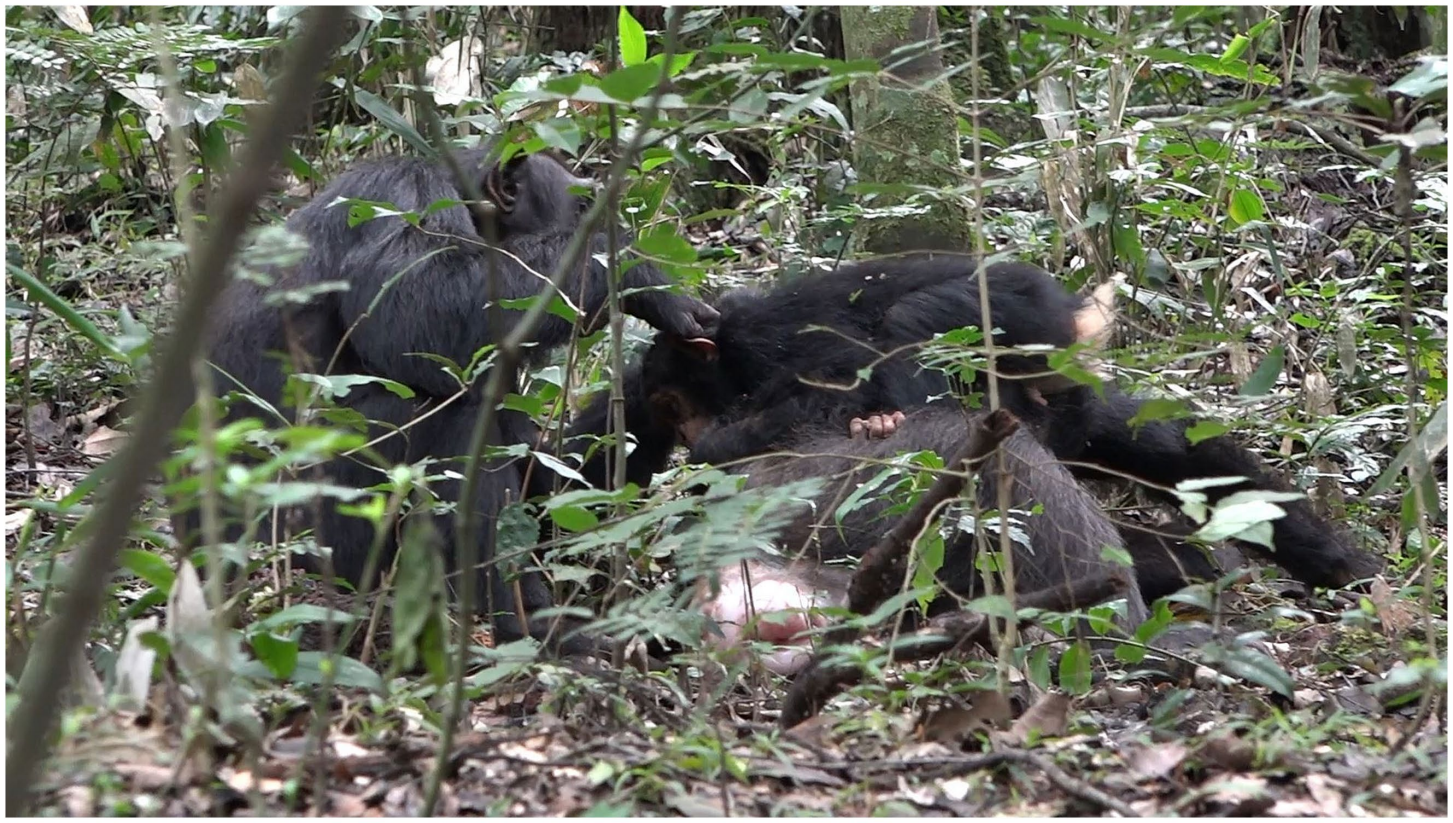
Episode 4: Non-aligning responding move (MOVE 2) of Williams (grooming Louis).

After ~5 s of grooming from Williams, Louis gets out of his mother’s embrace, sits up and starts climbing over his mother’s body toward Williams with his head lowered. He gets into a position where there is a participation framework between the two chimpanzees where contact social play would be possible, in the form, for example, of Williams tickling Louis’ lowered head (Figure 7A). When Louis reaches Williams, he brings his face close to Williams’ right hand and play bites it. He also lowers his head, and half opens his mouth in a PLAY FACE. As such, Louis has unilaterally initiated play (MOVE 1: Figure 7B). Williams, however, does not align with this movement toward joint contact social play; he physically engages with Louis (unlike the case in Episode 3) but rather than engaging in play with Louis, he engages in a different contact activity which Louis’ posture affords, i.e., grooming (MOVE 2). Williams changes his gaze, looking toward the top of Louis’ head, and places both his left and right hand there. He then replaces his right hand and moves Louis’ head down a bit while making grooming strokes with both hands (Figure 8). At this point, therefore, Louis and Williams are on different contact activity ‘agendas’, with the former engaging in play and the latter engaging in grooming. Grooming continues for ~3 s, and Louis then retracts his body away from Williams and ends his PLAY FACE. Louis returns back to Sabin’s chest and Williams restarts grooming Sabin. This episode shows that a participation framework has the ability to create intercorporeal affordances for more than one type of activity (for example, for both playing and grooming), with each chimpanzee engaging in a different activity.

### 3.2 Ending contact social play

We noted above that contact social play was initiated when an initiating move into play by one chimpanzee was responded to with an aligning move into play by another chimpanzee such that both were then simultaneously involved in that play. The end of a play bout (and thus potentially a play session if another bout is not initiated) involves the cessation of simultaneous joint play, most commonly by one chimpanzee disengaging from the play. This disengaging can take the form of still physically engaging with the other chimpanzee but now in the form of a non-play activity, or of stopping the play-related physical arrangement with the other chimpanzee without embarking on a joint non-play activity. We will examine an example of each of these in turn.

#### 3.2.1 Ending of a contact social play bout by one chimpanzee engaging in a joint non-play activity

**Figure 9:**
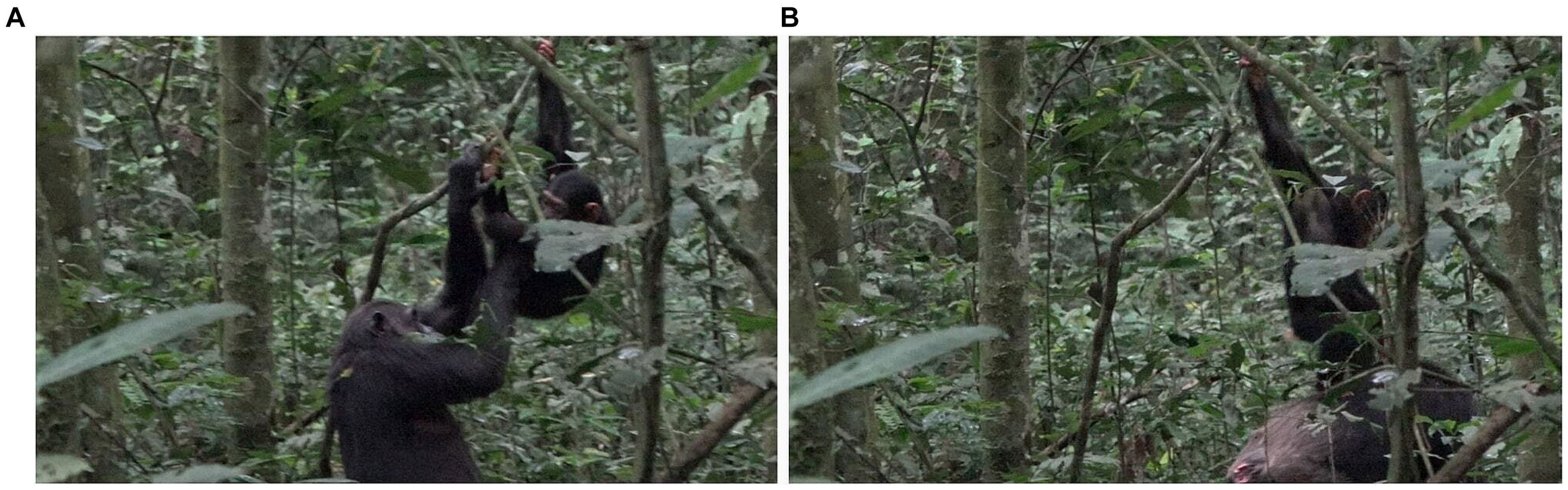
Episode 5: **(A)** Physical arrangement between E.O. (top) and Carson (bottom) in contact social play, **(B)** end of contact social play by Carson transitioning affordance for play to affordance for travel.

As we join this episode (Episode 5), the infant E.O. is engaging in contact social play with his mother Carson (Supplementary Video S5). This playing activity takes the form of E.O. hanging from a branch in front of, and facing, Carson at a level above Carson’s head. Carson plays by reaching up and tickling E.O. (Figure 9A). After ~4 s of this play, Carson turns to her right, gazing toward the researcher, and stops the tickling. This may be in response to hearing the researcher talk to his colleague. She then immediately starts to move away from the location, lowering her back in a manner which affords E.O. to climb onto it, and they leave the location together (Figure 9B). As such, the contact social play bout here is ended through Carson transitioning to a different, non-play, joint activity, i.e., leaving with E.O. on her back.

#### 3.2.2 Ending of a contact social play bout by one chimpanzee stopping play-related physical engagement, with no transition to another joint activity

**Figure 10:**
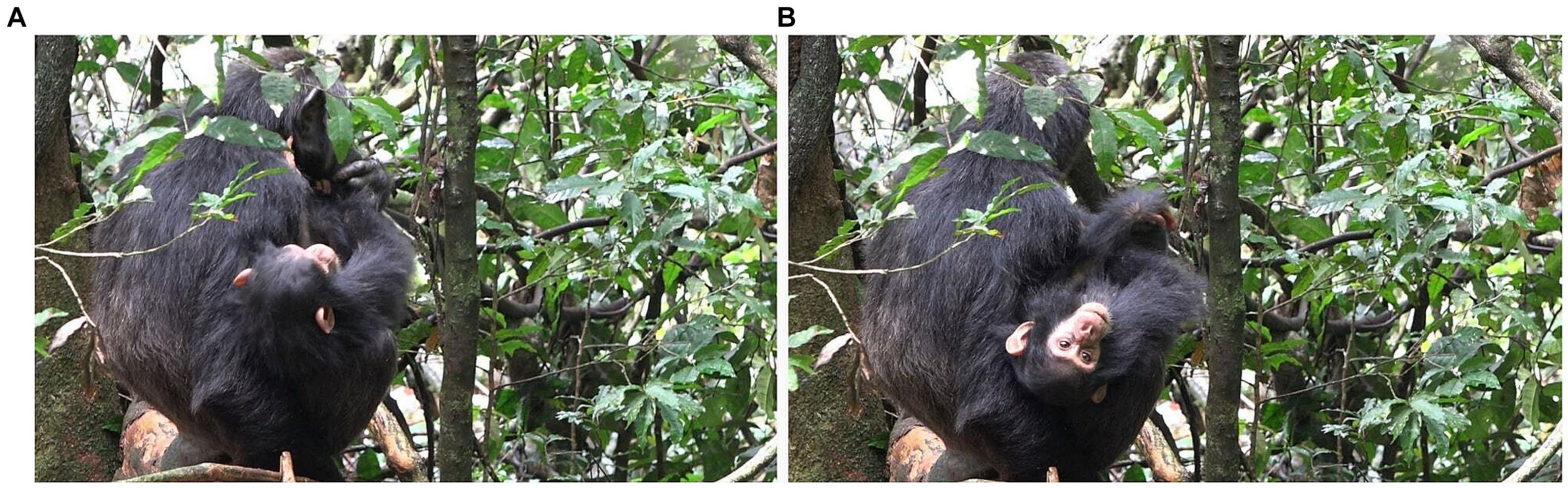
Episode 6: **(A)** Penelope play biting Lecter, **(B)** Penelope ends play-related physical arrangement by moving her head away.

As we join Episode 6, Penelope and Lecter are having a brief play bout, involving Penelope play biting Lecter (Supplementary Video S6). This play bout takes place ~23 s after the end of the dyad’s first play bout (whose initiation was described in Section 3.1.1.1). Lecter is once again lying in Penelope’s arms in a similar nested arrangement. Lecter has produced two MOUTH STROKE gestures in succession, and Penelope has reacted with a PLAY FACE and has started play biting Lecter’s hand (Figure 10A).

This play bout, however, only lasts a few seconds, as Penelope then grabs Lecter’s hand and removes it from her face, resulting in the play-related intercorporeal arrangement being broken. In addition, Penelope then turns her head away to her left, perhaps in response to noise nearby, and this diminishes the affordance for the type of play the dyad has just been engaged in, i.e., play biting (Figure 10B). Although Lecter is still nested passively in Penelope’s arms, affording further play-biting, Penelope continues to turn her head from side to side, looking around in response to noises nearby, and does not bring her mouth near to Lecter’s body again. Later play biting between Lecter and Penelope is reinitiated.

### 3.3 Maintaining contact social play

How contact social play is maintained can be examined in terms of (1) how a single *bout of play* is maintained after its initiation until its end, and also (2) how a *session of play* is maintained by moving from one bout to another. We examine both here. In the first case (Section 3.3.1), a question can be how the chimpanzees communicate/display to each other that the bout of play is ongoing and continuing. In the second case (Section 3.3.2), a question can be how one or both chimpanzees may communicate that despite a play bout having ended, one or both of them are available for another play bout, thus possibly extending the play session.

#### 3.3.1 Maintaining a play bout after initiation

**Figure 11:**
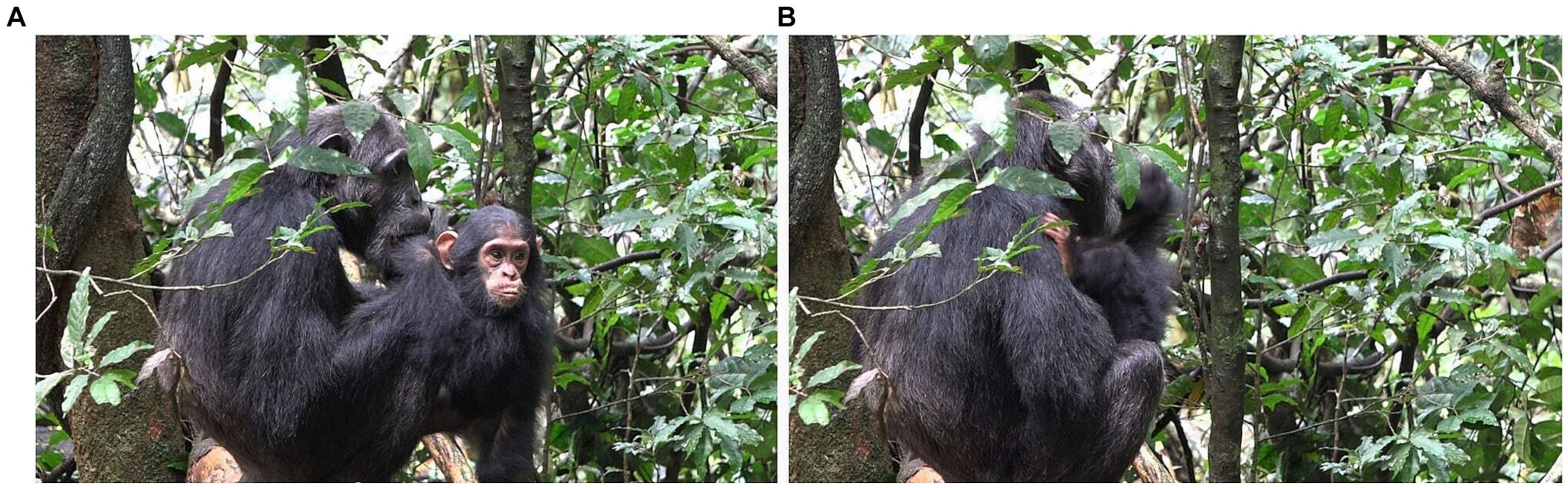
Episode 7: **(A)** Responding move (MOVE 2) of Penelope (play bite) initiating contact social play, **(B)** Lecter displaying alignment (MOVE3) by climbing into Penelope’s lap increasing the affordance for play biting.

**Figure 12:**
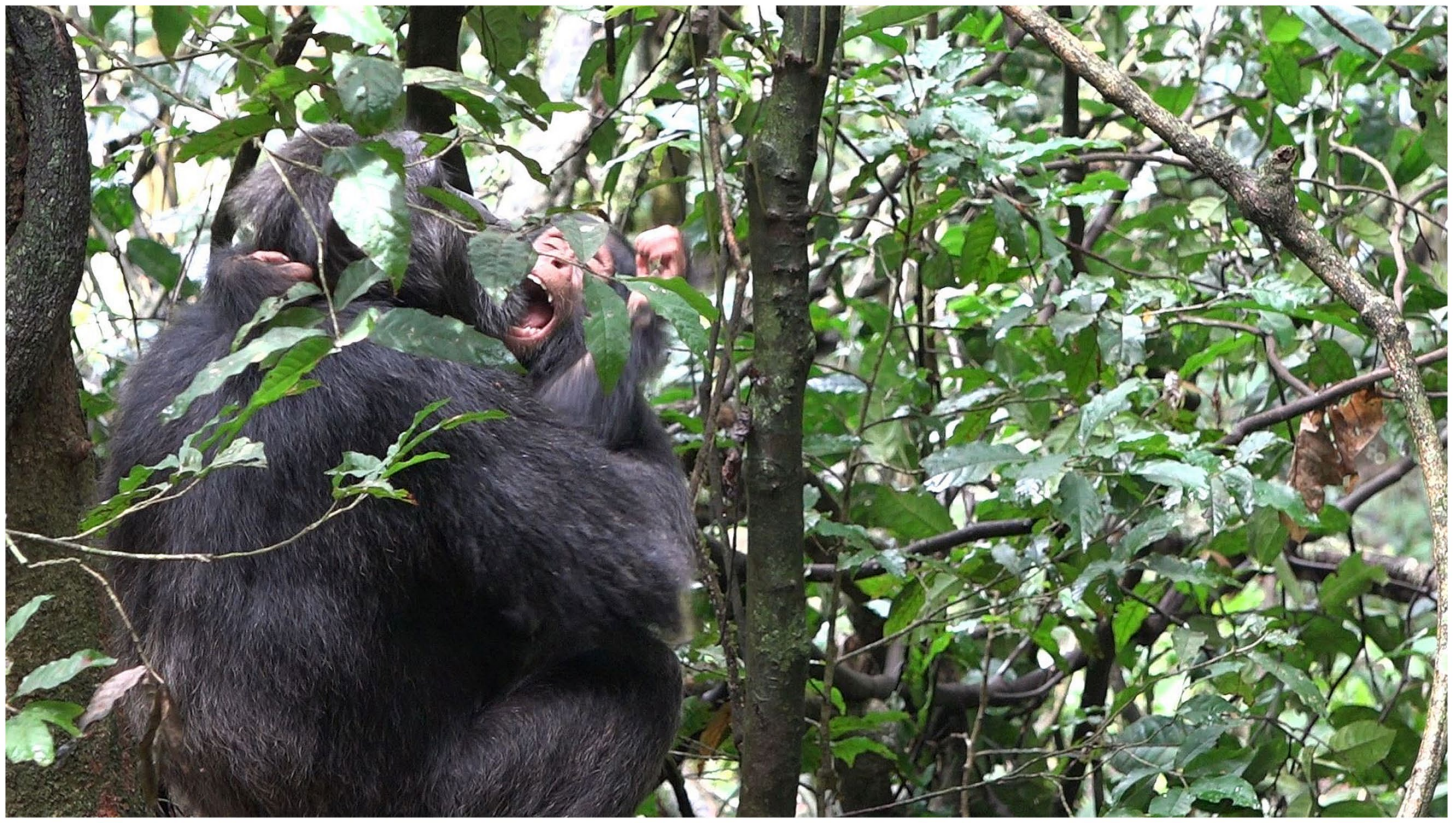
Episode 7: Continued contact social play by both Penelope (play biting) and Lecter (PLAY FACE).

In Episode 1 (Section 3.1.1.1), we followed Penelope and Lecter up to the point where they had started to engage in contact social play, after Lecter brought his left hand in front of Penelope’s mouth and held it there in a MOUTH STROKE gesture (MOVE 1) (Supplementary Video S7). Penelope went on to close her mouth on Lecter’s hand, play biting it (MOVE 2: Figure 11A). Since both Penelope and Lecter are in physical contact with each other, each is simultaneously acting on the other’s body, and the notion of discrete moves by either can be less relevant. Nevertheless, it is possible to see that Lecter next (MOVE 3) clambers across Penelope’s sitting body toward her left shoulder, getting into a position where his torso is lying in front of Penelope’s face, providing an affordance for Penelope to play bite it (Figure 11B). As such, it is possible to see that at the point of Lecter’s move (MOVE 3), the two chimpanzees are each displaying to the other that they are engaged together in the joint activity of social play. That is, when Lecter held his hand in front of Penelope’s mouth and used a MOUTH STROKE gesture, this could be understood by Penelope as a move into contact social play (MOVE 1). At that point she could have not engaged with Lecter (similar to what was seen in episode 3, Section 3.1.2.1) or engaged with him in another activity, such as grooming (similar to what was seen in episode 4, Section 3.1.2.2). But by aligning with Lecter’s move into play with a play move of her own, i.e., play biting (MOVE 2) she responded to that move as a move into social play, and displayed a willingness to go along with it. In turn, following Penelope’s MOVE 2, Lecter could similarly have not engaged with Penelope’s playing. However, by instead continuing to engage in a play-related manner (MOVE 3), he displayed a willingness to continue this particular course of action (contact social play) that the two had now launched. As such, by means of alignment across these three moves, Penelope and Lecter are implicitly displaying agreement (Schegloff, 1992) concerning what each wants and is willing to do at this point, i.e., to engage in contact social play (see, in contrast, episode 4, Section 3.1.2.2, where there is a form of ‘disagreement’ as to whether the activity that should be undertaken is playing or grooming). This agreement about the joint activity and a willingness to continue it is then further displayed by Penelope’s reaction to the affordance presented by Lecter’s body for play biting it by indeed engaging in play biting. The intercorporeal agreement about the joint activity underway (i.e., social play) continues with Lecter turning around on Penelope’s body and putting the side of his face next to her mouth. Penelope reacts by play biting Lecter’s face and Lecter displays a PLAY FACE (Figure 12). The bout ends when Penelope ceases to engage with the play by raising her face away from Lecter’s body in response to some surrounding noises and gazes in the direction from where the noises are coming.

#### 3.3.2 Maintaining a play session between play bouts

When a play bout ends, one or both chimpanzees may display that they are available for a further play bout (Supplementary Video S8). One way in which they can do this is by continuing to maintain their body in such a way that displays an affordance for play. This type of *affordance maintenance during play cessation* can be one factor in influencing whether or not the end of a contact social play bout will also be the end of the play session, with the affordance maintenance facilitating a further play bout and hence the continuation of the play session.

**Figure 13:**
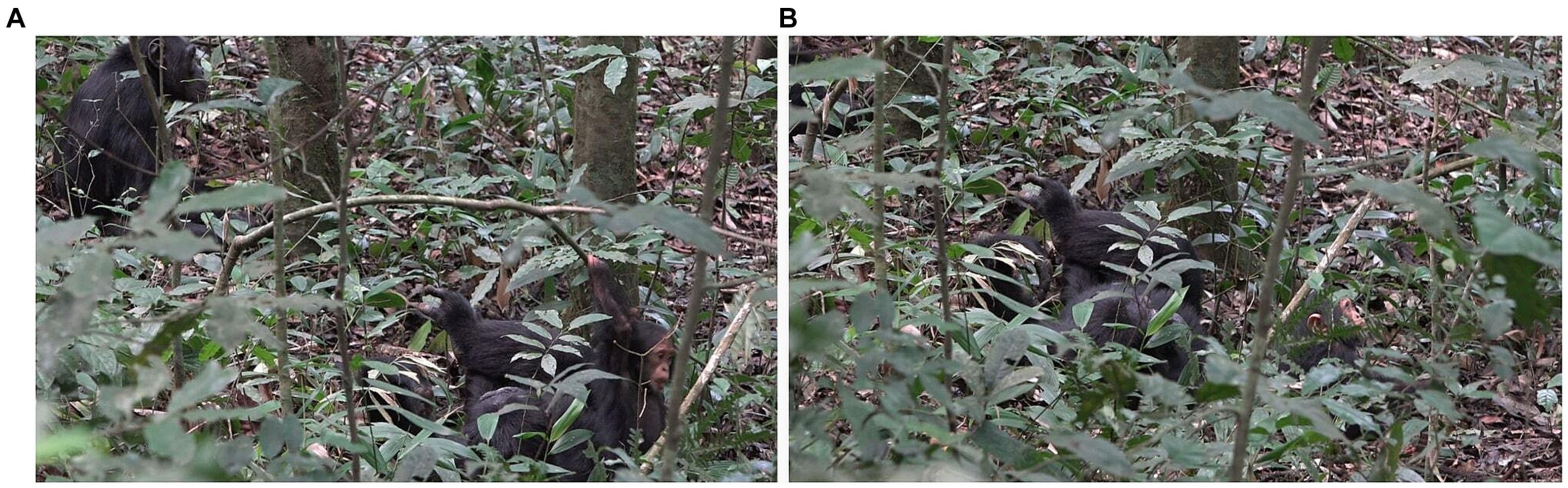
Episode 8: **(A)** Lootus (right) ends contact social play by moving off of Rollins’ body, **(B)** Rollins keeping up affordance for play as well as using gaze while Lootus (right) explores her surroundings.

**Figure 14:**
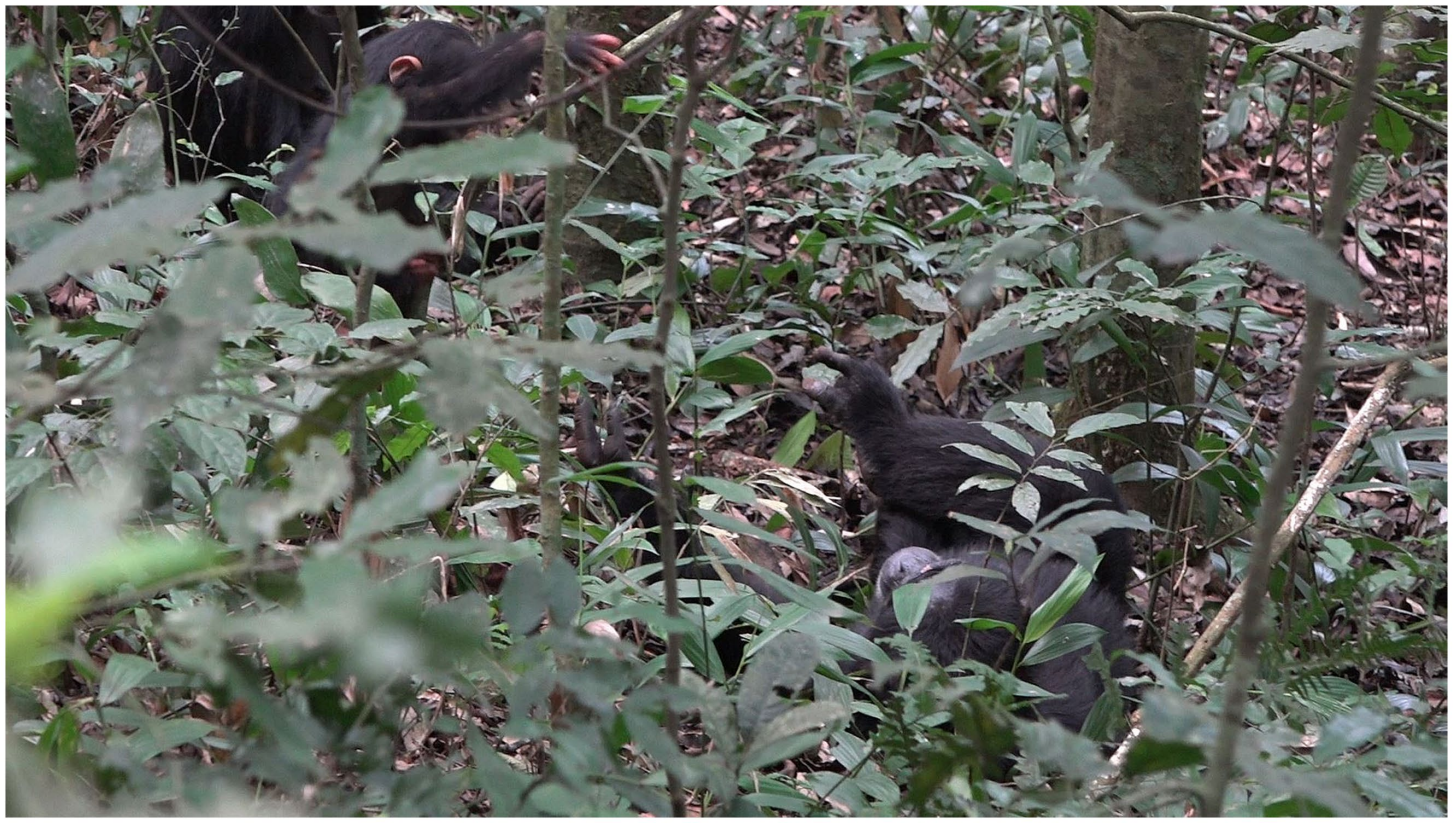
Episode 8: Collaborative recreation of participation framework where Lootus swings down onto Rollins.

In Episode 8, the play bout that we saw being initiated in Episode 2 (Section 3.1.1.2) has been brought to an end by Lootus stopping play-related physical engagement (involving Lootus hanging from the sapling onto Rollins’ reclining body, with Rollins patting her) by moving off of Rollins’ body (Figure 13A). As Lootus moves off his body and starts to explore the other sapling, Rollins maintains his reclining position. For the ~25 s between the two play bouts, while Lootus explores nearby, Rollins maintains his reclining body posture, and thus the affordance for further similar play (involving Lootus hanging from the sapling onto Rollins’ reclining body), gazing toward Lootus from time to time (Figure 13B). ~20 s after stopping the previous play bout, Lootus moves toward the original sapling again and starts climbing up. Rollins gazes at Lootus and as soon as she starts climbing performs an EXTEND HAND gesture while Lootus gazes at Rollins. The two bodies have now created a participation framework for further play (Figure 14) and Lootus then swings down onto Rollins and a new playing bout starts, with Rollins performing multiple GRAB gestures.

## 4 Discussion

The main aim of this paper was threefold: First, we wanted to show how adopting an intentionality-agnostic approach used in conversation analysis (Sidnell and Stivers, 2013) and other areas of interaction research can enrich the comparative study on communication. Second, we drew on insights from research into intercorporeality (Meyer et al., 2017; Meyer and von Wedelstaedt, 2017) and affordances (Gibson, 1986; Hutchby, 2001) to investigate communication and coordinated practical action between chimpanzees. Third, we applied the concepts of intercorporeality and bodily affordance to analyze how chimpanzees were able to achieve the rapid ‘on-the-fly’ coordination (Heesen et al., 2017) involved in a particular dynamic form of activity, i.e., contact social play between adults and infants, including how they were able to collaboratively achieve the initiation, ending and maintenance of that play.

The investigation has highlighted the important role played by the intercorporeal relationship between the chimpanzees’ bodies in achieving coordinated social play activities. This includes how, within the ongoing activity, one chimpanzee’s body can provide a bodily affordance, setting up an opportunity for another chimpanzee to produce a responsive action and thus potentially move the activity forward in a coordinated manner. We have explored how, in ways such as this, a joint activity, such as contact social play, can be co-constructed on a move-by-move basis by the two chimpanzees. As such, we have aimed to show how chimpanzee contact social play does not rely solely, or even perhaps primarily, on signals (intentionally produced or not) and gaze; rather, as can be seen in human activities, such as dancing or sport (Meyer et al., 2017; Meyer and von Wedelstaedt, 2017), bodies can coordinate together drawing on resources such as bodily affordances and an understanding of the structure of the activity. By using the concepts of intercorporeality and bodily affordances any joint activity can be analyzed from the bottom up focusing on how its different phases (opening, main body, closing: Heesen et al., 2017) are structured. On top of that “bottom layer” of bodies interacting, interactants might make use of more complex intentional signals and gaze patterns.

### 4.1 Future directions

The examples used in this paper have shown that the concepts of intercorporeality and bodily affordance are useful for the analysis of social play interactions between infant and adult chimpanzees. However, these concepts can easily be used more broadly across interactions in general. While the notion of bodily affordances has clear applicability in capturing features of a dynamic, physical activity such as contact social play, it is by no means limited to that type of activity. A recent article of Mondada and Meguerditchian (2022) used a comparable approach to study greetings in Olive baboons (*Papio anubis*) showing how some formats of action create different affordances warranting different responses. Another joint action that has been suggested as a context of interest is grooming (Genty et al., 2020; Heesen et al., 2021a). A commonly used signal in grooming is the “present” gesture defined as an individual offering a body part such as the arm, armpit, back, genital region, leg, or rump to the recipient for subsequent grooming (Pika, 2014). At the same time, “present” is a perfect example of creating the affordance for the next step in the interaction: the grooming of that specific body part (Hobaiter and Byrne, 2011; Roberts et al., 2014). Numerous examples can be given in other situations as well (e.g., a mother performing the “lower back” gesture to afford for her infant to climb on in order to leave together; Fröhlich et al., 2016b). These examples also highlight a way in which bodily affordances can explain the use of specific gestures in certain contexts. While not every gesture might be explainable in terms of bodily affordances (e.g., “leaf clipping”: Nishida, 1980; Boesch, 1995; Nakamura et al., 2000; Pika and Deschner, 2019), the framework does add another dimension in which the production, usage and meaning of gestures can be studied and possibly better understood. Gestures could then be measured based on how much their meaning – derived from the ASO method (Hobaiter and Byrne, 2014) – overlaps with the affordance the gesture creates. As can be seen from Episode 4, some movements can create multiple conflicting affordances and in these cases, intentionally produced signals could help disambiguate the situation (Leavens et al., 2005; Roberts et al., 2013; Roberts and Roberts, 2022). Future studies may investigate if the production of intentionally produced signals is dependent on the existence of simultaneous produced conflicting bodily affordances.

The present paper has shown that while bodily affordances and gestures are based on different communication paradigms (dynamic system theory vs. information-processing), both can be studied simultaneously and that they reinforce each other, giving a more complete description of the whole interaction. As can be seen in the episodes, participants made use of both signals and bodily affordances to achieve coordinated joint activity. This has shown that the concepts of intercorporeality and bodily affordance add to the study of animal communication but do not replace more “traditional” measurements even though there is overlap between them, where, for example, gestures themselves create bodily affordances. Another direction in which the present framework may be taken is the study of the role played by bodily affordances in the acquisition of gestures (Hutchins and Johnson, 2009). Gestures may be shaped by the affordances that they represent. This would fit the social negotiation hypothesis which postulates that gestures emerge from a repeated exchange of social behaviors between interactants (Pika and Fröhlich, 2019). Interactants might initially make use of bodily affordances but over repeated exchanges a negotiation of certain behaviors might lead to the co-creation of gestures. If that is the case, one could expect gestures where the meaning is close to the affordance they create to appear earlier in development. Additionally, this convergence of signals negotiated by both participants could be augmented by intentionality markers on top of the basis of bodily affordances (Leavens and Hopkins, 1998; Pika and Bugnyar, 2011; Roberts and Roberts, 2019; Schel et al., 2022). However, these lines of enquiry are outside the scope of the current paper and will not be pursued further here.

In our results, we showed the role of bodily affordances in the opening, main body and exit phase of social play following the joint action framework of Heesen et al. (2017). As these phases also appear in playing interactions in humans (Clark, 1996; Clark, 2006; Rossano et al., 2022) future research could investigate the role of bodily affordances comparatively.

In addition, the notion of intercorporeality and bodily affordance is species-agnostic. Unlike more traditional approaches, bodily affordances can be studied independent from the cognitive mechanisms that underlie them. This allows for bodily affordances to be studied in any species in the animal kingdom that shows social behavior involving two bodies interacting with each other. Species that are similar in build and anatomy will create similar bodily affordances. Taking this approach will thus greatly increase possibilities for cross-species comparative research.

### 4.2 Limitations

With extracts from only four different play interactions, this paper works with a limited dataset. Therefore, any patterns shown here might not represent general patterns in playing interactions in chimpanzees in the wild. For example, a common play signal laughter, did not occur in any of our examples (Van Hooff, 1972; Matsusaka, 2004; Ross et al., 2010). Additionally, confounding factors, such as social bond or rank and age difference that might influence the playing interaction were not analyzed (Heesen et al., 2020, 2021a,b). However, the aim of this paper was to introduce the concepts of intercorporeality and bodily affordance to the field of comparative communication research and demonstrate how these could be used to analyze dynamic chimpanzee social interactions. Therefore, this paper should be viewed as a “proof of concept” and not as evidence for general patterns of bodily affordance employed by all chimpanzees.

Following previous literature, we defined the end of a playing session as the cessation of play behavior for more than two minutes (Heesen et al., 2021a,b). However, ultimately, using time criteria to classify between an interruption and a true ending is arbitrary. Stronger evidence of whether the participants experienced the joint activity as being ended could be found in the follow-up behavior of the participants. An interruption might be characterized by at least one participant attempting to actively reinitiate a new playing bout or passively keeping up the affordance for play (e.g., Episode 8), whereas a true ending might be characterized by both participants breaking the participation framework and “moving on” to another (joint) activity (Episode 5). In three out of four of the extracts analyzed in this paper, the joint activity of social contact play was ended by one or both participants engaging in a joint-travel interaction (e.g., Episode 5), thus physically removing themselves from the place of play and severely diminishing the optionality of the previously established type of play restarting. In the fourth extract (Episode 4) the option for re-engagement of play remained as the physical arrangement did not significantly change after the episode. However, there was no successful initiation of social contact play to start from and, additionally, following this unsuccessful initiation, both participants did not engage with each other for at least three minutes after the analyzed episode.

While the concepts of intercorporeality and bodily affordance can be used very flexibly, they also have their limitations. As the names suggest, intercorporeality and bodily affordance deal with physical movements and physical arrangements of bodies. Therefore, they will not add a new dimension to the study of purely vocal interactions such as long-distance vocal exchanges (Arcadi, 2000; Geissmann, 2002; Schamberg et al., 2016; Southern et al., in preperation). However, most communicative interactions are inherently multimodal (but see for behavioral ecologists: Partan and Marler, 1999; Hebets and Papaj, 2005), with multimodal referring to the simultaneous or sequential integration of signals from at least two ‘modalities’ (Liebal et al., 2013; Luef and Pika, 2017). As can be seen in the present paper, bodily affordances can be analyzed in conjunction with other signals of different modalities.

### 4.3 Implication for the study of language evolution

Levinson’s interaction engine hypothesis states that certain characteristics of social communicative interactions may have preceded the evolution of language and the different components of this “interaction engine” might have different evolutionary origins (Levinson, 2006). A crucial window onto these evolutionary origins and different antiquities is the comparative approach, enabling a comparison of communicative toolkits across closely-related species and beyond (e.g., Pika, 2008; Pika and Bugnyar, 2011). Comparative studies into communicative interactions may then focus on all components involved and not just on a subset of elements based on implied cognitive mechanisms such as intentionality. By *a priori* selecting specific parts based on intentionality criteria only a part of the communicative interaction is analyzed. Communicative interactions can contain many semiotic resources that are not captured by “traditional” measurements such as intentional signals and gaze. By drawing on the concepts of intercorporeality and bodily affordances, we showed that the common dynamic interaction of social play can be better understood through focusing on aspects of the activity which are typically glossed over, thus offering a new more complete window onto how joint activities in the animal kingdom are coordinated. We have aimed to demonstrate how chimpanzees coordinate contact social play through a form of sequential organization, using the intercorporeal relations between their bodies. This includes making use of the affordances these bodies provide, to coordinate activity on a move-by-move basis, where one move (intentionally produced or not) can set up a sequential ‘slot’ for the next move potentially to occur, and move the activity forward. This echoes findings about how human participants shift postures together, apparently almost simultaneously, where “close inspection of these moments usually shows that they involve sequential interaction in which one party proposes or preenacts a shift and is then, in a second step, joined by the other when he completes the change in posture” (Meyer et al., 2017, p. xxiii).

The sequential organization of bodily action is one aspect of the social organization of interaction which is evident in both human and nonhuman animals. It provides an apparent continuity between them in relation to how social activities are cooperatively co-constructed, with one participant monitoring the other, and producing conduct which can be seen by those present as responding to, and potentially aligning with, the other’s prior conduct. As Streeck (2017, p. 352), noted, to understand the evolution of human language and talk-in-interaction, it would appear to be important “to search for the primordial intercorporeal capacities from which the distinctly human form of sociality, language use in interaction, has emerged.” The concepts of intercorporeality and bodily affordance may therefore allow the start of broader comparisons involving carefully chosen representatives of primates and beyond. It allows us to study every type of interaction that involves bodies. From this bottom layer of interacting bodies, we can stack evidence of more “intentional” signals and gaze, to make comparisons between human interactions and those of other animals in terms of complexity. In this paper, we have shown that animals can communicate by doing instead of just by signaling and moved away from a too narrow focus on intentionality only. Therefore, the approach that we have highlighted here might bring us one step closer to finally solving the puzzle that is the evolution of language and may in addition open up other pathways in the field of comparative psychology.

## Data availability statement

The original contributions presented in the study are included in the article/Supplementary material, further inquiries can be directed to the corresponding author.

## Ethics statement

The present study was purely observational and non-invasive. All applicable national, and/or institutional guidelines for the care and use of animals were followed. In accordance with the German Animal Welfare Act of 25th May 1998, Section V, Article 7, the study was classified as non-animal experiment and did not require any approval from a relevant body. All observers followed a strict hygiene protocol, including a seven-day quarantine, and wore face masks when encountering chimpanzees. Observations were made at a minimum distance of seven meters, in an effort to avoid disease transmission from humans to chimpanzees (Leendertz et al., 2004; Köndgen et al., 2008) and to not disturb the natural behaviour of the chimpanzees observed. Our research adhered to the legal requirements of the state of Uganda and was approved by the by the Ugandan Wildlife Authority, and the Ugandan National Council for Science and Technology. It followed the recommendations of the ‘Animals (Scientific Procedures) Act 1986’, as published by the government of the United Kingdom, and the principles of “Ethical Treatment of Non-Human Primates”, as stated by the American Society of Primatologists.

## Author contributions

BB: conceptualization, data collection, analysis, writing – original draft, and writing – reviewing and editing. RW: conceptualization, analysis, and writing – reviewing and editing. SP: conceptualization, funding acquisition, supervision, and writing – reviewing and editing. All authors contributed to the article and approved the submitted version.
