# Supplementary material for: Bodies at play: the role of intercorporeality and bodily affordances in coordinating social play in chimpanzees in the wild
Source: Front Psychol. 2024 Jan 16;14:1206497. doi: 10.3389/fpsyg.2023.1206497 (PMC10826840; doi:10.3389/fpsyg.2023.1206497)
Supplement: Supplementary file 1 [file Data_Sheet_1.docx]

Supplementary Material

Bodies at play: The role of body postures and their affordances in coordinating play in chimpanzees in the wild

Bas van Boekholt^1, *^, Ray Wilkinson^2,^ and Simone Pika^1^

^1^ Comparative BioCognition, Institute of Cognitive Science, University of Osnabrück, Osnabrück, Germany

^2^ Division of Human Communication Sciences, Health Sciences School, University of Sheffield, Sheffield, United Kingdom

*** Correspondence:**Corresponding Author
[basvanboekholt@hotmail.com](mailto:basvanboekholt@hotmail.com)

**Supplementary material**

Table S1: Detailed information about the used videos

| **Episodes** | **Infant** | **Age in years (Estimated date of Birth)** | **Adult (relation with infant)** | **Age in years (Estimated date of Birth)** | **Date of video** |
| --- | --- | --- | --- | --- | --- |
| 1, 6 and 7 | Lecter | 1.8 (04-11-19) | Penelope (mother) | 30.7 (01-01-91) | 05-09-21 |
| 2, 3 and 8 | Lootus | 1.1 (26-07-20) | Rollins (unrelated) | 35.7 (01-01-86) | 12-09-21 |
| 4 | Louis | 3.6 (25-01-18) | Williams (unrelated) | 13.9 (01-10-07) | 02-09-21 |
| 5 | E.O. | 3.3 (05-12-17) | Carson (mother) | 27.0 (05-04-94) | 04-04-21 |

Video S1: Penelope & Lecter, initiating contact social play involving play biting

<https://youtu.be/CuMEqH66Jsg>

Video S2: Rollins & Lootus, initiating contact social play involving falling and patting

<https://youtu.be/5fLiBFPmLHA>

Video S3: Rollins & Lootus, unilateral initiation of social play which did not lead to joint initiation due to chimpanzee B not engaging with chimpanzee A

<https://youtu.be/zyEh6sPBI1c>

Video S4: Williams & Louis, unilateral initiation of social play which did not lead to joint initiation due to chimpanzee B engaging with chimpanzee A but with a non-play activity

<https://youtu.be/b_86jSb7u5c>

Video S5: Carson & E.O., ending of a contact social play bout by one chimpanzee engaging in a joint non-play activity

<https://youtu.be/IxyBCRIKbKo>

Video S6: Penelope & Lecter, ending of a contact social play bout by one chimpanzee stopping play-related physical engagement, with no transition to another joint activity

<https://youtu.be/6oud8NLHbS8>

Video S7: Penelope & Lecter, maintaining a play bout after initiation

<https://youtu.be/1O-wTLgz7g0>

Video S8: Rollins & Lootus, maintaining a play session between play bouts

<https://youtu.be/-R7YRSmKehM>
